# Society Meetings

**Published:** 1893-03

**Authors:** 


### Bristol Medico-Chirurgical Society.

*January 11th*, 1893.

Dr. Shingleton Smith in the Chair.

Dr. Watson Williams described the case of a boy who for five years had expectorated daily large quantities of pus. He had cavernous breathing, and amphoric voice-sounds from the third to the fifth spaces in the axillary line, with other signs of a cavity. An incision was made by Dr. J. Swain, but no definite cavity could be found, although pus drained away later. The boy died soon after, and at the autopsy the lung was found to be full of small cavities separated by dense fibrous tissue. These had given rise to physical signs simulating an abscess.

Dr. Prowse showed sections of a liver with great dilatation of the bile ducts and numerous cavities, due to the breaking down of cancerous deposit. The specimens were from a woman, aged fifty-eight, who had suffered from attacks of hepatic colic with the passage of gall-stones.

Mr. Barfield Adams showed the brain of a boy, aged eleven, who had suffered from loss of vision, nystagmus, and extensive inco-ordination of the limbs. The head had considerably increased in size. The patient had been in the Infirmary, where pressure on the cerebellar region had been diagnosed, and the operation of tapping proposed, but this was not allowed by the friends. After death a spherical cyst was found between the lateral lobes of the cerebellum. The ventricular cavities were much distended. There was also a small cystic dilatation of the infundibulum and pituitary body. A committee was appointed to investigate the nature of the growth.

Mr. Dobson exhibited—(1) A periosteal sarcoma of the humerus, for which amputation at the shoulder was performed. (2) Numerous hard, bony bodies removed from the knee joint; they were covered in the recent state with cartilage. Some were free and some fixed. (3) A large ulcerated tumour of the breast, involving the pectoralis major. It had been growing for a year.

---

*February 8th*, 1893.

Dr. E. Markham Skerritt, President, in the Chair.

Dr. Harrison and Dr. Waldo exhibited some interesting cases of skin disease, including—(1) Several examples of psoriasis. (2) Iodide of potassium rash in a syphilitic subject. (3) Cases of partial and universal alopecia. (4) Folliculitis barbæ. (5) Lupus-like rash, which was probably syphilitic, &c.

Mr. Morton read a report drawn up by the committee appointed to investigate Mr. Adams's case of cerebellar cyst. A small gliomatous tumour was found in connection with the larger cyst. He also showed a specimen of villous tumour of the bladder.

Mr. Pickering showed two specimens of tubercular kidney. The first was removed by nephrectomy from a woman aged thirty. Nephrotomy was performed in September, 1891, but a sinus remained which discharged urine and pus; consequently the entire kidney was removed in July, 1892. She made a good recovery. The second specimen was from a patient who did not show definite signs of kidney disease during life.

Dr. EDGEWORTH read a paper on the motor fibres of the cranial nerves, in which the results of an anatomical investigation (the expenses of which were defrayed by the Royal Society) were applied to elucidate some obscure clinical phenomena. The following points were especially dwelt on: the innervation of the orbicularis oris, of the digastric, of the levator palati, and of the laryngeal muscles.

---

*March 8th*, 1893.

Dr. E. MARKHAM SKERRITT, President, in the Chair.

Mr. HARSANT exhibited an ulcerated vermiform appendix he had recently removed by operation from a boy. The patient made a good recovery.

Dr. EDGEWORTH showed a boy with a firm, solid swelling in both hypochondriac regions, reaching on the right side nearly to Poupart's ligament. It was thought to be tubercular thickening of omentum.

Dr. MICHELL CLARKE showed a boy and a girl, the subjects of hereditary ataxy. In one of them the knee-jerks were increased. The chief features of this condition were pointed out.

Dr. PROWSE read notes of a case of coma occurring in a young married woman. She had suffered for some weeks from severe abdominal pain, with alternate diarrhœa and constipation, and occasional delirium. She became semi-conscious, and this condition deepened into coma. The urine was healthy, but contained a quantity of lithates and crystals of uric acid. The pulse was very quick. There was no suppression of urine. At the autopsy, a small hemorrhage, the diameter of a sixpenny-piece, was found in the right lenticular nucleus. The uterus contained twins. Kidneys healthy, but cortex slightly diminished. The large intestine was studded over with small bodies like miliary tubercles. Microscopic examination had not clearly revealed the nature of these deposits. The other organs were healthy.

Dr. H. MACLEOD gave a demonstration of various bacteria by means of the oxy-hydrogen lantern and photographs. The bacilli of tetanus, cholera, diphtheria, &c., were shown.

Mr. W. DUNCAN gave the history of a case of a primipara, aged 25, who had given birth to two healthy children, each child being expelled from a distinct uterine cavity. The two uteri could be felt contracting separately, and the septum between the two could be detected.

Dr. ROGERS brought before the Society the case of a female patient who had for six years suffered from myxœdema, attended with the usual symptoms. She was treated by 20-minim doses of Brady and Martin's extract of thyroid, with marked success. Improvement in speech had begun five days after commencing the treatment.

Dr. MICHELL CLARKE showed (1) a specimen of great cystic degeneration of the kidneys; and (2) an aortic aneurism which had perforated into the œsophagus, causing sudden death. There were few symptoms during life.

G. MUNRO SMITH, *Hon. Sec.*

